# Seeing Bradycardia: How Ultrasound Improves Medical Decision-Making

**DOI:** 10.7759/cureus.65951

**Published:** 2024-08-01

**Authors:** Jared L Cohen, Amie Billstrom, Melissa Myers

**Affiliations:** 1 Emergency Department, San Antonio Military Medical Center, San Antonio, USA; 2 Emergency Department, Brooke Army Medical Center, Fort Sam Houston, USA

**Keywords:** bradycardia, symptomatic bradycardia, echocardiography, regional wall motion abnormalities, ultrasound (u/s), clinical decision support, non-st segment elevation myocardial infarction (nstemi), av node dysfunction, cardiac echo

## Abstract

There is a broad differential for new-onset cardiac dysrhythmia, and the rapid identification of the underlying cause of these cardiac emergencies can be lifesaving. Identifying wall motion abnormalities on point-of-care ultrasound (POCUS) is not a core echocardiography application for Emergency Medicine (EM) physicians. However, ruling in a regional wall motion abnormality can expedite patient-centered care and assist the busy EM physician in high-risk cases.

## Introduction

An atrioventricular (AV) block signifies abnormal signal propagation between the atria and the ventricle and is a common cause of bradycardia in the Emergency Department (ED). Up to 20% of patients suffering an acute myocardial infarction (AMI) develop an AV conduction abnormality, and ischemic heart disease accounts for approximately 40% of all AV blocks [1]. These conduction abnormalities post-AMI have an increased mortality rate as well [2]. Medications are also common culprits of AV blocks presenting to the ED [3]. Invasive procedures, such as open heart surgery, transcatheter aortic valve replacement [4], catheter ablation for arrhythmias, transcatheter closure of ventricular septal defects (VSDs), and alcohol septal ablation are also common causes for new AV conduction abnormalities [5].

Patients with AV blocks may be completely asymptomatic or may present with varying degrees of fatigue, dyspnea, chest pain, syncope, or sudden cardiac arrest. The presence of the AV nodal blockade limits the appropriate physiologic response to acute changes in volume status or systemic vascular resistance. The subsequent bradycardia can cause a reduction in cardiac output as well as severely restricting one’s ability to augment the heart rate to meet one’s metabolic demand. Performing a point-of-care echocardiogram can provide insight into the patient's ejection fraction, volume status, and shock state of the patient. If a wall motion abnormality is noticed, ischemic causes of the heart block rise to the top of the differential. Obtaining this data at the bedside on patient presentation can positively impact patient-centered decision-making as is demonstrated through the following case presentation.

## Case presentation

A 75-year-old man presented to the ED with three days of generalized weakness. The patient reports that he was globally weak when he woke up three days ago and had difficulty completing activities of daily living. On the day of his presentation, he stated that he became lightheaded and had to crawl to the bathroom to avoid losing consciousness. He denied chest pain, shortness of breath, vomiting, diarrhea, or any recent illness. He complained of spending most of the day on the ground due to feeling weak. On patient presentation, he had a blood pressure of 168/94 and a heart rate of 32. Other vital signs were within normal limits. The patient was asymptomatic while lying in bed but became lightheaded upon movement of his extremities while in bed.

**Figure 1:**
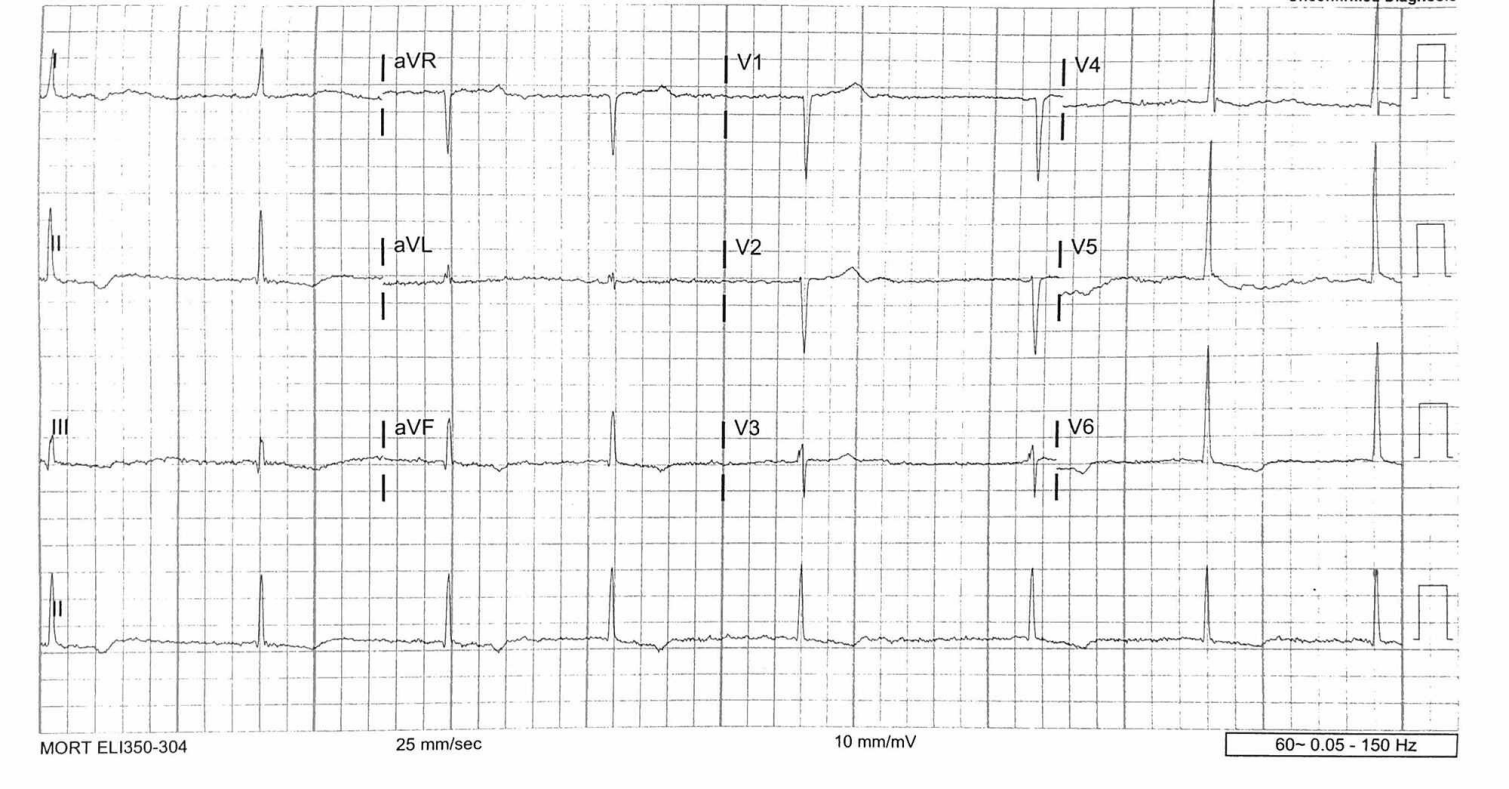
EKG

**Figure 2:**
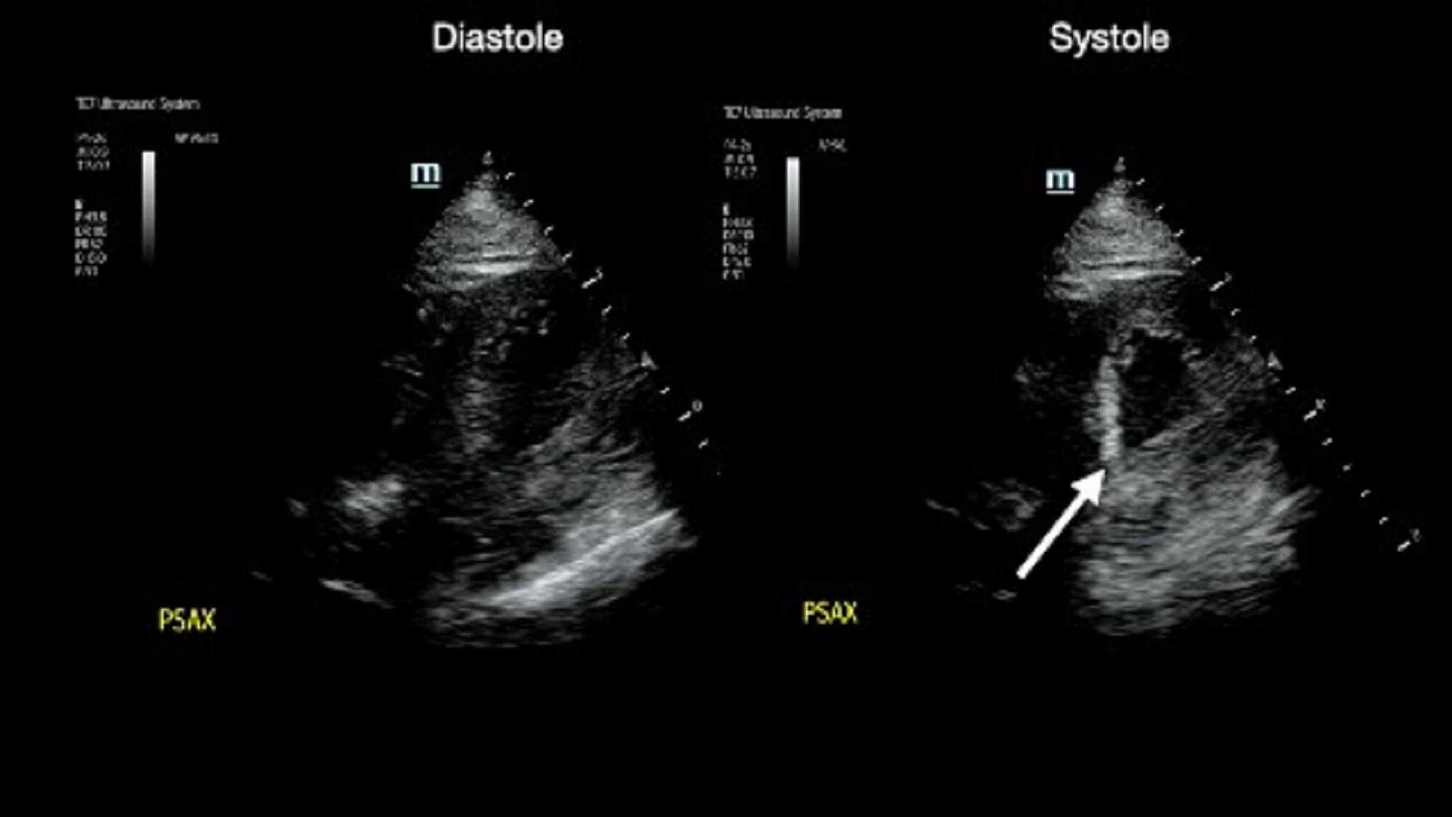
Symptomatic bradycardia wall motion abnormality: systole vs. diastole

The patient’s physical exam revealed an obese male without any focal finding on exam besides the bradycardia and movement-induced lightheadedness. An EKG showed slow atrial fibrillation with an irregular rate of 42 (Figure 1). A high-grade AV block was present, and there were Twave inversions in leads II, III, and aVF, as well as I, V6, and V5, without any ST-segment elevations or depressions (Figure 1). A cardiac point-of-care ultrasound was performed upon the patient's arrival and revealed a moderately reduced ejection fraction with inferior wall hypokinesis (Video 1, Figure 2).

**Video 1:** Symptomatic bradycardia wall motion abnormality

Cardiology was consulted shortly after the patient's arrival with concern for ischemic heart disease as a contributor to the patient’s symptomatic bradycardia. The patient’s troponin returned one hour after the patient arrived at 1.16 ng/mL (normal reference range < 0.04 ng/mL). The patient’s brain natriuretic peptide (BNP) was also elevated at 6,279 pg/mL (normal reference range < 100 pg/mL), and the remainder of his labs included a complete blood count, complete metabolic panel, thyroid stimulating hormone, and urinalysis, which were within normal limits. The patient was admitted to the Cardiac Intensive Care Unit for early invasive intervention on his non-ST segment elevation myocardial infarction (NSTEMI) as well as dual-chamber pacemaker placement.

## Discussion

Timely use of point-of-care echocardiography allowed for the rapid identification and management of an NSTEMI. Without the use of ultrasound, the time to diagnosis would have been delayed until the troponin resulted, and diagnostic uncertainty between a type 1 and type 2 NSTEMI would have still existed.

To evaluate for regional wall motion abnormalities, cardiologists check 17 different segments within the left ventricle. Emergency Medicine (EM) physicians will typically only grossly evaluate for wall motion abnormalities in three segments: the anterior, the lateral, and the inferior wall of their parasternal short-axis view [6]. These myocardium segments correlate with specific vascular territories: the left anterior descending, the circumflex, and the right coronary artery, respectively. A regional wall abnormality on POCUS may be the first clue to diagnosing an NSTEMI. The American Cardiology Association (ACA) and American Heart Association (AHA) guidelines for the management of unstable angina and NSTEMI recommend early invasive strategies for patients at elevated risk (Class 1A recommendation), with one of those risk factors identified as an abnormal finding on Echo [7].

While ruling out a regional wall motion abnormality is thought to be out of scope for many EM physicians, EM physicians have an 88% sensitivity and 92% specificity for diagnosing regional wall hypokinesis [8]. Ruling in regional wall motion abnormalities has utility in assisting in high-stress, high-liability cases. Diagnosing a new regional wall motion abnormality on POCUS can expedite patient care and allow the EM physician to advocate for the appropriate, early interventions in a time-sensitive manner. It both improves the time to the diagnosis and reduces diagnostic uncertainty in this scenario.

## Conclusions

Early cardiac POCUS may improve the time to diagnosis and the time to patient-centered treatment in the evaluation and treatment of emergent cardiac pathology. POCUS assists in classifying a patient's cardiac injury as a type 1 vs. type 2 NSTEMI and is useful in a comprehensive but rapid evaluation of emergent cardiovascular illness. In this example, an EM physician detected an unexpected cause of a new AV nodal block within minutes of the patient's presentation, leading to a streamlined management plan for a critically ill patient.
